# Back Pain as a Clue to Spinal Cord Involvement in Sjögren's Syndrome: An Uncommon Case

**DOI:** 10.7759/cureus.77999

**Published:** 2025-01-26

**Authors:** Ana Patrícia Moreira, Mafalda Vasconcelos, André Santos, Hugo Alves, João Espírito Santo

**Affiliations:** 1 Internal Medicine, Hospital Beatriz Ângelo, Loures, PRT

**Keywords:** central nervous system, longitudinally extensive transverse myelitis, meningomyeloradiculitis, paraparesis, sjögren's syndrome

## Abstract

Sjögren's syndrome (SS) is an autoimmune disease with systemic manifestations. Neurological manifestations of SS include vasculitis, dorsal ganglionitis, demyelination, myelitis, myeloradiculitis, meningoencephalitis, cognitive dysfunction, and autonomic dysfunction mediated by antibodies. We present a case of a 65-year-old woman with a significant medical history of rheumatoid arthritis and secondary Sjögren's syndrome, diagnosed 20 years prior, and treated non-Hodgkin lymphoma. She presented to the emergency department with progressive lower back pain, radiating to the lower limbs for the past five months, along with sensory loss, weakness in the lower limbs, urinary retention, fecal incontinence, and recurrent fever.

On examination, she had grade 4 paraparesis predominantly in the proximal muscles, with suspended thermoalgesic sensation from D6-11 and areas of allodynia in L5-S1, abolished deep tendon reflexes in the lower limbs, and absent bilateral plantar reflexes. MRI revealed a longitudinally extensive spinal cord lesion (from D4 to the conus medullaris) with T2 hyperintensity, contrast enhancement, and meningeal and cauda equina root thickening. Lumbar puncture showed lymphocytosis, hypoglycorrhachia, and hyperproteinorrachia, suggestive of lymphocytic meningitis. Microbiology, immunophenotyping, anti-aquaporin-4 antibodies, angiotensin-converting enzyme (ACE), calcium, and urinary calcium levels were normal. Meningeal biopsy at D10 showed an unspecific mononuclear inflammatory process. She received pulses of methylprednisolone but continued to experience paraparesis, fever, asthenia, and new vertebral joint arthritis/synovitis at L4-L5, leading to the initiation of cyclophosphamide treatment with progressive improvement. Sjögren's syndrome has a wide range of manifestations and can involve multiple systems. Spinal cord involvement is rare and presents diagnostic dilemmas between infectious causes, other autoimmune diseases, and neuromyelitis optica spectrum disorders.

## Introduction

Sjögren’s syndrome (SS) is a chronic autoimmune disorder primarily targeting the exocrine glands, leading to lymphocytic infiltration and inflammation. It can be present alone, which is known as primary Sjögren's syndrome (pSS), or with other systemic autoimmune disorders, known as secondary Sjögren's syndrome (sSS). The most common associated diseases are rheumatoid arthritis (RA) and systemic lupus erythematosus (SLE). It is most commonly associated with xerostomia and xerophthalmia, but the disease often extends beyond these hallmark symptoms to affect multiple organ systems, including musculoskeletal, renal, pulmonary, hematological, and particularly the nervous system. Neurological involvement, while less common, plays a significant role in the disease’s clinical presentation, affecting up to 20% of SS patients [1]. Peripheral nervous system (PNS) manifestations are well-documented and occur in approximately 10-20% of patients with primary SS. Central nervous system (CNS) involvement is often underreported. Typically, sicca symptoms appear about five years before CNS involvement.

Non-compressive myelopathies encompass a range of conditions, with neuromyelitis optica (NMO) being one of the most notable due to its characteristic presentation of longitudinally extensive transverse myelitis (LETM). LETM is a spinal cord lesion that spans three or more vertebral segments and is centrally located. NMO, often involving optic neuritis and severe spinal cord damage, is the most common cause of LETM and a key feature in its diagnostic criteria. However, other diseases, such as acute disseminated encephalomyelitis (ADEM), spinal cord infarction (or other vascular myelopathies), and autoimmune-related conditions such as SLE, sarcoidosis, SS, antiphospholipid syndrome, mixed connective tissue disease, scleroderma, ankylosing spondylitis, Behcet’s disease, and sarcoidosis can also present with LETM [2].

The primary differentiating features between NMO and other non-compressive myelopathies include the extent and severity of spinal cord involvement and the presence of aquaporin-4 antibodies, which are typically positive in NMO but negative in other conditions. In contrast, lesions in other myelopathies usually involve fewer than two vertebral segments, can present bilaterally, and are less severe.

The clinical features of transverse myelitis (TM) include acute or subacute (over 4 h to four weeks) sensory and autonomic involvement related to spinal cord lesions. It presents with intense pain in the neck or between the shoulder blades followed by sensory and motor deficits below the site of injury. In subacute and chronic forms, symptoms often include sensory disturbances, urinary incontinence, and difficulty walking, which can progress to spastic paraplegia. The cervical and upper thoracic regions are most frequently affected. Subacute cases are linked to urinary problems, optic neuritis, and possible paralysis. Cerebrospinal fluid studies may reveal spinal inflammation, with elevated white cells or IgG levels. Recurrence of TM is associated with anti-Ro/SSA antibodies [3].

Longitudinally extensive transverse myelitis (LETM) is a spinal cord lesion that spans three or more vertebral segments and is centrally located. Neuromyelitis optica (NMO) is the most common cause of LETM, typically involving optic neuritis and spinal cord damage. LETM is a key feature of NMO and part of its diagnostic criteria. However, other autoimmune diseases like multiple sclerosis, acute disseminated encephalomyelitis, SLE, sarcoidosis, and SS can also present with LETM. Proper evaluation is crucial to differentiate the different etiologies and exclude diseases, such as infections, cancers, vascular diseases, nutritional deficiencies, or traumatic injury. Identifying the exact cause is essential for determining the appropriate management, treatment, and prognosis [4]. We present a case of LETM in a patient with secondary SS, highlighting the importance of considering this diagnosis in patients with SS who develop neurological symptoms.

This study was previously presented as a manuscript at the VII Congresso Nacional de Autoimunidade - XXVI Reunião Anual do NEDAI in 2021.

## Case presentation

We present a case of a 67-year-old black woman with a history of rheumatoid arthritis overlapping with Sjögren's syndrome diagnosed 20 years prior, along with heart failure, hypertension, type 2 diabetes, and Hodgkin lymphoma treated in 1995, without recurrence. She was on chronic medication with 5 mg of prednisolone. The patient presented to the outpatient clinic at the beginning of February with complaints of progressive lower back pain radiating to the lower limbs, which had worsened over the previous three to four months, requiring multiple emergency department (ED) visits due to uncontrolled pain at home.

Since January, the patient reported perianal sensory loss, progressing to thigh involvement. In early February, she experienced a single episode of fecal incontinence, accompanied by difficulty in local muscle contraction. Subsequently, her pain extended to the left hemithorax, with associated lower limb weakness, gait instability, and difficulty maintaining an upright position.

Given these symptoms, she was referred to the ED and admitted for further investigation. On examination, she exhibited grade 4 paraparesis, predominantly proximal, with impaired thigh flexion. Osteotendinous reflexes were absent and Babinski sign was present. While vibratory sensation was preserved, she demonstrated reduced pain and temperature sensation at the T6-T11 dermatomes on the left side, allodynia along the lateral aspect of the leg, and bilateral hypoesthesia along the S1 level of both feet. Laboratory tests only showed slightly elevated liver enzymes (Table 1).

**Table 1:** Investigation profile of the patient at the time of admission.

Investigations	Patient	Reference values
Hemoglobin	14 g/dL	12-16 g/dL
Total leukocyte count	7,870/dL	4,000-10,000/dL
Platelet count	196,000/dL	150,000-400,000/dL
Serum creatinine	0.81 mg/dL	0.5-1.2 mg/dL
Aspartate aminotransferase	41 U/L	<32 U/L
Alanine aminotransferase	47 U/L	<33 U/L
Alkaline phosphatase	67 U/L	35-105 U/L
Gamma-glutamyl transferase	46 U/L	6-42 U/L
C-reactive protein	0.24 mg/dL	<0.5 mg/dL

**Figure 1:**
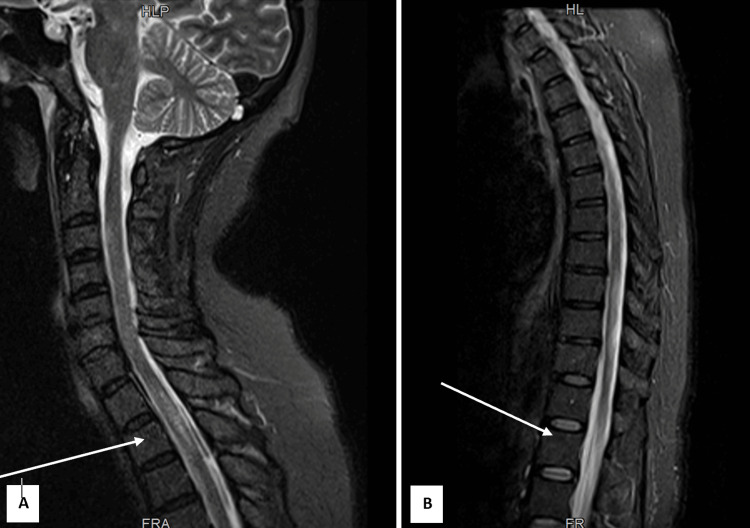
Sagittal magnetic resonance imaging scans at admission. The images (A) cervicodorsal region and (B) thoracolumbar region show a T2 hyperintense signal extending from T4 to the conus medullaris, predominantly central in distribution (indicated by arrows with greater intensity).

This clinical presentation suggested a subacute or chronic myelitis or radiculitis. A spinal computed tomography (CT) scan ruled out a compressive lesion. An urgent MRI was performed which also showed meningeal thickening resembling a sleeve, with abnormal enhancement post-contrast, and diffuse thickening of the cauda equina nerve roots, suggesting multifocal meningeal, medullary, and radicular involvement (Figures 1A, 1B). The differential diagnoses included tuberculosis, lymphoma, and autoimmune etiologies (considering the patient’s known autoimmune diseases or another, such as sarcoidosis). There was no intracranial involvement.

A lumbar puncture revealed cerebrospinal fluid (CSF) pleocytosis with a predominance of mononuclear cells, marked hypoglycorrhachia (19% of glycemia), and elevated protein levels. Cytology was consistent with lymphocytic meningitis, and immunophenotyping excluded monoclonality, ruling out lymphoma. Direct tests on CSF (for fungi, *Mycobacterium tuberculosis*, and bacteria) were negative, as were cultures from blood and CSF. PCR testing for *Mycobacterium tuberculosis *DNA was negative (mycobacterial cultures as well).

Extensive serological testing in both peripheral blood and cerebrospinal fluid (CSF) was negative for Brucella, Leptospira, Mycoplasma, Epstein-Barr virus, Cytomegalovirus, and Coxsackie virus. Sarcoidosis was deemed unlikely due to normal angiotensin-converting enzyme levels, normal calcium levels, and unremarkable findings on thoracic CT.

Antinuclear antibodies (ANA) were positive at a titer of 1:160 with a fine granular pattern, though ANA was negative in the CSF. Complement levels were normal. Anti-SSA and anti-SSB antibodies were negative, as were tests for ANCA, lupus anticoagulant, β2-glycoprotein, anti-cardiolipin, and anti-aquaporin 4.

A follow-up MRI showed similar meningeal enhancement and new medullary lesions. Given the chronic meningomyeloradiculitis without an identified etiology despite extensive investigation, the case was evaluated by neurosurgery for a meningeal biopsy, which was performed at the beginning of April, without complications. The biopsy revealed a non-specific, sparse mononuclear inflammatory process. Following the biopsy, the patient was started on intravenous methylprednisolone (1 g/day for three days). The patient underwent five cycles of cyclophosphamide and was discharged. Pain management and physiotherapy were implemented during the hospitalization, leading to improvements in walking ability. At discharge, the patient showed the following signs: paraparesis (muscle strength grade 4 in thigh flexion bilaterally, grade 5 distally in the right lower limb, and grade 4+ distally in the left lower limb); the patellar deep tendon reflex was absent on the left and diminished on the right; distal hyperesthesia in the lower limbs extending up to the knees; a suspended level of mild hypoesthesia to thermal and pain sensation from T5 to T11 on the right and T6 to T11 on the left, with a saddle-shaped area of hypoesthesia; plantar reflexes were absent. Gait was possible with bilateral support.

**Figure 2:**
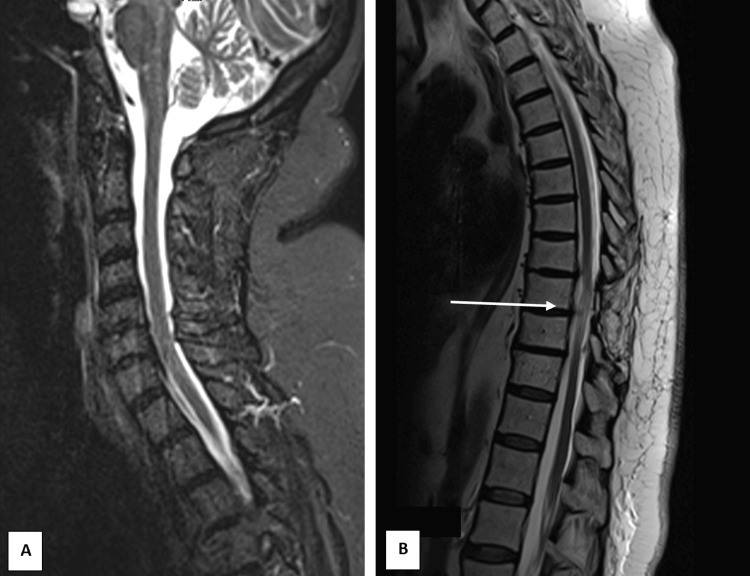
Sagittal magnetic resonance imaging scans six months after treatment. The images (A) cervicodorsal region and (B) thoracolumbar region demonstrate residual signal enhancement at the site of the bilateral laminectomy defect at the T9 level (arrow), consistent with chronic changes. No additional abnormalities are noted.

On follow-up as an out-patient, the MRI was repeated in August and no other abnormal enhancements were identified, including intramedullary or leptomeningeal, only showing evidence of residual changes due to the meningeal biopsy (Figures 2A, 2B). The patient recovered full mobility with continued physical therapy.

## Discussion

The described case underscores significant aspects of SS, particularly the potential severity and complexity of its neurological manifestations, with a focus on CNS involvement. While PNS complications are better documented, occurring in 10-20% of patients with pSS, CNS involvement remains less frequent and its true prevalence is not well established. A study noted a higher prevalence of PNS involvement in sSS compared to pSS (31% vs. 19%, p<0.05), while CNS involvement showed similar rates (13% vs. 15%) in both subtypes [5].

The mechanisms underlying CNS involvement in SS are incompletely understood but likely multifactorial. Vasculitic processes, analogous to those affecting other organs in SS, are thought to play a major role. Pathological studies have demonstrated perivascular mononuclear cell infiltration, microinfarcts, microhemorrhages, and endothelial damage in CNS tissue. In some cases, autoimmune demyelination has also been implicated, adding another layer of complexity to the disease’s neurological manifestations [5,6].

CNS involvement in SS can present a broad spectrum of symptoms, including seizures, cognitive impairment, aseptic meningoencephalitis, acute or chronic progressive myelopathy, and spinal subarachnoid abnormalities. Specific spinal cord-related conditions, such as TM, chronic progressive myelopathy, and even rare syndromes like Brown-Séquard syndrome, further complicate the clinical presentation [6]. TM, a significant manifestation in both pSS and sSS, involves focal or segmental inflammation of the spinal cord, often resulting in severe neurological deficits.

This is one of the most recognized neurological complications in SS, although it remains rare. It is more frequently associated with sSS, particularly in cases secondary to SLE or rheumatoid arthritis [7]. LETM is a hallmark of autoimmune causes (as explained before), including SS, and often leads to significant disability due to extensive involvement of motor, sensory, and autonomic tracts.

The clinical presentation of TM includes paraparesis or quadriparesis, sensory deficits, urinary retention, bowel dysfunction, and, frequently, radicular or localized back pain, as seen in our patient. Systemic inflammatory symptoms, such as fever and arthritis, may accompany the neurological findings, necessitating careful differential diagnosis from infectious myelitis, NMO spectrum disorder, and other autoimmune conditions [3].

Diagnosis of TM in SS relies on imaging, CSF analysis, and serological testing. MRI typically reveals T2 hyperintensities in the spinal cord, often with gadolinium enhancement indicating active inflammation. CSF findings may include lymphocytic pleocytosis and elevated protein levels, as found in our patient, but also occasionally oligoclonal bands [8]. Serological testing for anti-SSA and anti-SSB antibodies, as well as markers for associated autoimmune conditions like SLE, aids in confirming the diagnosis and excluding mimickers.

CNS involvement in SS, although rare, poses significant diagnostic challenges due to its overlap with other autoimmune and infectious diseases. Early recognition is essential, as delayed diagnosis can result in irreversible neurological damage. This case illustrates the diverse and potentially severe neurological manifestations of SS, highlighting the need for heightened clinical awareness and a multidisciplinary approach. Further research is required to elucidate the true prevalence of CNS involvement in SS and optimize management strategies to improve outcomes and quality of life for affected patients.

## Conclusions

The study concludes that CNS manifestations are a rare but possible phenomenon in severe Sjögren's syndrome. As presented, the case report shows a patient who developed transverse myelitis as a central nervous system manifestation of Sjögren's syndrome. The prompt diagnosis and initiation of cyclophosphamide led to the resolution of the transverse myelitis. This study illustrates the importance of early diagnosis and management of such a manifestation.
